# Morphological Evaluation of Tumor-Infiltrating Lymphocytes (TILs) to Investigate Invasive Breast Cancer Immunogenicity, Reveal Lymphocytic Networks and Help Relapse Prediction: A Retrospective Study

**DOI:** 10.3390/ijms18091936

**Published:** 2017-09-08

**Authors:** Gloria Romagnoli, Meike Wiedermann, Friederike Hübner, Antonia Wenners, Micaela Mathiak, Christoph Röcken, Nicolai Maass, Wolfram Klapper, Ibrahim Alkatout

**Affiliations:** 1 School of Life & Health Sciences, Aston Brain Centre, Aston University, Birmingham B4 7ET, UK; 2 Department of Radiology and Neuroradiology, Klinikum Dortmund, 44137 Dortmund, Germany; meikewiedermann@googlemail.com; 3 Department of Gynecology and Obstetrics, University Hospitals Schleswig-Holstein, 24105 Campus Kiel, Germany; rike_huebner@gmx.de (F.H.); Antonia.Wenners@gmx.de (A.W.); Nicolai.Maass@uksh.de (N.M.); 4 Fertility Center Kiel, 24103 Kiel, Germany; 5 Department of Pathology, General Pathology and Hematopathology, University Hospitals Schleswig-Holstein, 24105 Campus Kiel, Germany; Micaela.Mathiak@uksh.de (M.M.); christoph.roecken@uksh.de (C.R.); wklapper@path.uni-kiel.de (W.K.)

**Keywords:** breast cancer, tumor-infiltrating lymphocytes (TILs), tumor heterogeneity, tumor immunogenicity, CD3, CD8, FoxP3, regulatory T cells (Tregs), CD8/FoxP3 ratio, CD3/FoxP3 ratio

## Abstract

Tumor-infiltrating lymphocytes (TILs) in breast cancer are a key representative of the tumor immune microenvironment and have been shown to provide prognostic and predictive biomarkers. The extent of lymphocytic infiltration in tumor tissues can be assessed by evaluating hematoxylin and eosin (H&E)-stained tumor sections. We investigated tissue microarrays of 31 invasive breast cancer patients, looking at quantity and topological distribution of CD3+, CD8+, CD20+, Ki67+, FoxP3+ TILs and CD3+/FoxP3+, CD8+/FoxP3+ cell ratios. We separately evaluated TILs at the invasive edge and at the center of the tumor, to find any clinical implications of tumor heterogeneity. No statistically significant difference was found in quantity and distribution of both TIL subsets and TIL ratios, by comparing patients who suffered from a local or distant recurrence of the tumor (relapse group: 13 patients) with patients not showing cancer relapse (non-relapse group: 18 patients). In the whole sample, we observed three main statistically significant positive correlations: (1) between CD3+ and CD8+ T-cells; (2) between FoxP3+ and Ki67+ lymphocyte infiltration; (3) between CD3+/FoxP3+ cell ratio (C3FR) and CD8+/FoxP3+ cell ratio (C8FR). Tumor heterogeneity and stronger positive TIL associations were found in the non-relapse group, where both CD3–CD8 and FoxP3-Ki67 inter-correlations were found to be significant at the center of the tumor, while the correlation between C3FR and C8FR was significant at the invasive edge. No correlations between TIL subsets were detected in the relapse group. Our findings suggest the existence of stronger inter-subtype lymphocytic networks in invasive breast cancer not showing recurrence. Further evaluations of clinical and topological correlations between and within TIL subsets are needed, in addition to the assessment of TIL quantification and distribution, in order to follow up on whether morphological evaluation of TILs might reveal the underlying lymphocytic functional connectivity and help relapse prediction.

## 1. Introduction

Breast cancer accounts for 25% of all cancers in developed countries [1] and is the first cancer world-wide affecting women, at a mean age of 64 years [2]. Apart from tumor staging and grading, only a few reliable prognostic factors, such as hormone receptor and HER2 expression, are currently available for breast cancer, to estimate the chance of disease recovery or relapse. New biomarkers of risk and prognosis are therefore needed to guide and improve therapies toward a successful clinical outcome.

The approach to identify new prognostic biomarkers is complex, because it must look at the composite scenario of tumor progression and all its determinants, such as the critical interplay between cancer cells and the immune microenvironment. Ever since Virchow (1863) and Paget (1889) pointed out a connection between chronic inflammation and cancer development, the importance of the immune microenvironment for cancer cell proliferation has gained more and more attention [3,4]. Today, it is possible to monitor the tumor immune microenvironment by looking at the tumor-infiltrating lymphocytes (TILs), which control tissue homeostasis and the activation of innate and adaptive immune cells [5]. TILs are widely considered to be a key indicator of the immune interaction between host and tumor, and potentially effective predictive biomarkers of cancer immunogenicity, clinical outcome, response to immunotherapy and other antitumor treatments [5,6,7,8,9,10,11]. Although lymphocytic infiltrates have long been observed in breast cancer, only recent clinical trials have demonstrated the immunogenic nature of breast cancer and the potential role of host immunosurveillance in influencing tumor progression and treatment responses [5,8,12,13,14,15,16,17,18,19,20,21,22,23,24]. B-cell infiltrates seem to play only a minor role in mammary tumor, where CD20+ cells are sporadically detected [25,26]. In contrast, macrophages and T-cells are very likely to be found within breast tumor, as TILs, as well as in the surrounding stroma, as stromal tumor-infiltrating lymphocytes (STILs) [20]. Nevertheless, the actual function of lymphocytic infiltrations is still debated, with several studies reporting discrepant results [5,27,28,29,30]. It is thought that TILs play dual roles in cancer, by either suppressing or helping the immune responses; their prognostic impact is further complicated by molecular subtypes and immune system variability [31]. On one hand, “suppressor” TIL subsets (e.g., FoxP3+, CD4+) can harbor immunosuppressive activity, promote tumor invasion and restrict the effectiveness of immunotherapeutic strategies [29,30]; on the other hand, “effector” TILs (e.g., CD3+, CD8+) have substantial anti-tumor and anti-proliferative capabilities, and have been found to be associated with improved pathological response and better clinical outcome [5,18,22,32,33,34,35,36,37,38].

The present study is intended to complement our previous investigations on epithelial-to-mesenchymal transition (EMT) markers and cancer stem cells (CSCs) in normal breast tissue and invasive breast cancer [39], and on the prognostic significance of Snail and FoxP3 in invasive ductal breast cancer [31]. In those works, we already stated the existence of immunoactive cells (CD3+, CD8+ and FoxP3+) in our cohort of patients, recognizing the further need to better determine whether they may have an impact as prognostic biomarkers [31,39]. To this end, in the current work we are going to characterize, quantify and investigate distribution and inter-/intra-correlations of TIL subpopulations, in the same cohort of patients affected by invasive breast cancer [39].

By using morphological evaluation of TILs as main tool, we are seeking to reveal the lymphocytic networks underpinning tumor immune microenvironment, and shed new light on their function and prognostic impact.

## 2. Results

### 2.1. Quantification and Distribution of TIL Subsets

**Figure 1:**
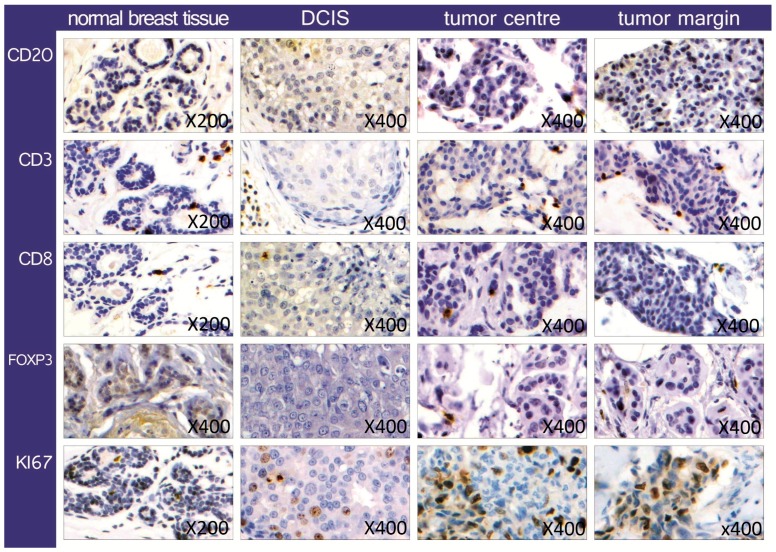
Representative examples of the immunohistochemical staining of the lymphocyte markers CD20, CD3, CD8, FoxP3, Ki67, for normal breast epithelium, DCIS and invasive breast cancer, the latter divided in tumor center and margin.

We used antibodies that allowed us to identify the invasion of different types of lymphatic cells in a breast cancer cell cluster. The pan-keratin antibody helped to identify all breast cancer cells and distinguish them from stromal cells. A positive staining for the surface marker CD20 showed all B-lymphocytes, while CD3 marked all T-lymphocytes. The cytotoxic T-cells were identified by CD8 staining, while a positive reaction with a FoxP3 antibody showed only the regulatory T-cells (Tregs). The Ki67 is a marker of cell proliferation, often correlated to cancer clinical course. This combination of antibodies made it possible to detect the lymphatic cells in the cancer, identify them and get information about their distribution and quantity. Lymphatic cells were found in the cancer as well as in normal tissue, but TILs in normal breast tissue and ductal carcinoma in situ (DCIS) were not counted. Representative examples are shown in Figure 1.

There is currently no evidence showing whether TILs at the tumor edge functionally differ from those located in the inner stroma, and to which extent tumor heterogeneity might be clinically relevant in breast cancer [20]. In light of this, we evaluated TILs at the invasive edge as a separate parameter from TILs in the tumor center, to investigate clinical implications of breast tumor heterogeneity, in patients showing local or distant relapse of the tumor as well as in those without tumor recurrence.

In an invasive cancer formation, 0.15% B-lymphocytes (CD20+) were detected, while there were 3.78% T-cells (CD3+). Looking at the breast cancer infiltration of B-cells and T-cells, no statistically significant difference between tumor center and margin was found (CD3 *p*-value 0.263, CD20 *p*-value 0.127). Similarly, no statistically significant difference was observed in the distribution of cytotoxic T-cells (CD8+) and Tregs (FoxP3+) between inner stroma and invasive edge of the tumor (CD8 *p*-value 0.409, FoxP3 *p*-value 0.232). Only few CD20+ cells and FoxP3+ cells were identified in both invasive cancer and normal breast tissue, so that it was not always possible to take pictures with internal positive controls (see Figure 1). Moreover, there was no statistically significant difference in the incidence of all lymphatic cell types between the relapse and non-relapse group (*p*-values for: CD3 0.825, CD8 0.137, CD20 0.447, FoxP3 0.801). Similarly, the Ki67 marker showed a homogeneous distribution of the proliferating cells, since the proliferation rate was found not to be significantly different neither comparing center to margin of the tumor (*p*-value 0.580) nor relapse to non-relapse group (*p*-value 0.753).

Other sensitive indicators for monitoring immune function within tumor microenvironment are the ratios of immune effector T cells (CD3+ and CD8+) to immune suppressor T cells (FOXP3+): CD3+/FOXP3+ and CD8+/FOXP3+. Therefore, we analyzed the CD3+/FOXP3+ ratio, and found no statistically significant difference neither between tumor margin and center (*p*-value 0.298) nor between the relapse and non-relapse group (*p*-value 0.886). Similarly, looking at the CD8+/FOXP3+ ratio, no significant difference was observed neither between tumor edge and center (*p*-value 0.524) nor between relapse and non-relapse group (*p*-value 0.334). The expression of all markers in invasive breast cancer tissues is summarized in Table 1.

### 2.2. Topological and Clinical Correlations between Different TIL Subsets

**Figure 2:**
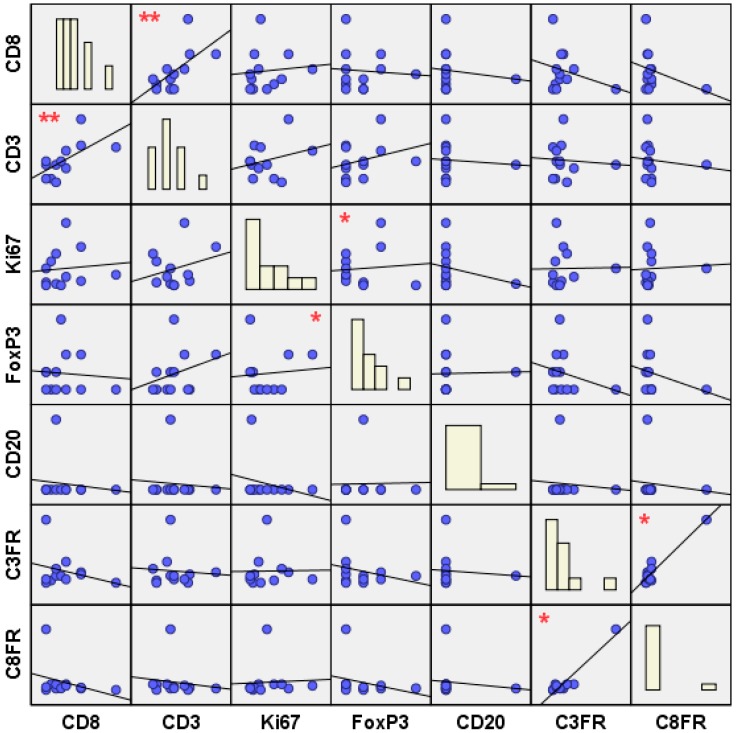
Scatterplot matrix (SPLOM) of correlations betweendifferent TIL subsets (CD8+, CD3+, Ki67+, FoxP3+, CD20+), CD3+/FoxP3+ (C3FR) and CD8+/FoxP3+ (C8FR) cell ratios. Histograms of the variables are shown in the diagonal. Only for SPLOM purposes, missing values were excluded listwise, to obtain a consistent case base for the chart. * Correlation is significant at the 0.05 level (2-tailed). ** Correlation is significant at the 0.01 level (2-tailed).

While looking for possible associations between different TIL subsets (inter-subtype correlations), in the whole sample we observed three statistically significant positive correlations, as shown in Figure 2: (1) between CD3+ and CD8+ lymphocyte infiltration (*r* = 0.392, *p* = 0.009); (2) between FoxP3+ and Ki67+ lymphocyte infiltration (*r* = 0.337, *p* = 0.024); (3) between CD3+/FoxP3+ (C3FR) and CD8+/FoxP3+ (C8FR) cell ratios (*r* = 0.560, *p* = 0.013).

Analyzing these correlations across topological groups, we further observed: (1) a significant positive correlation between CD3+ and CD8+ TILs at the tumor center (*r* = 0.496, *p* = 0.031); (2) a significant positive correlation between FoxP3+ TILs at the tumor center and Ki67+ TILs both at the tumor center (*r* = 0.803, *p* = 0.000) and margin (*r* = 0.457, *p* = 0.043); (3) a significant positive correlation between C3FR and C8FR at the invasive edge (*r* = 0.884, *p* = 0.000).

Moreover, we analyzed the same correlations across the clinical groups and subgroups, observing: (1) a significant positive correlation between CD3+ and CD8+ TILs in the non-relapse group (*r* = 0.469, *p* = 0.016); (2) a significant positive correlation between FoxP3+ and Ki67+ TILs in the non-relapse group (*r* = 0.550, *p* = 0.003), where, in particular, Ki67+ TILs at the tumor center were found to be positively correlated with both FoxP3+ TILs at the tumor center (*r* = 0.887, *p* = 0.000) and FoxP3+ TILs at the tumor margin (*r* = 0.582, *p* = 0.037); (3) a significant positive correlation was found between C3FR and C8FR at the margin of the tumor not showing relapse (*r* = 0.911, *p* = 0.004).

### 2.3. Topological and Clinical Correlations within TIL Subsets

While looking for possible associations within TIL subsets (intra-subtype correlations) across the topological groups, we observed that CD3+, Ki67+ and FoxP3+ TILs at the tumor center were positively correlated with their respective subsets at the tumor margin (CD3+ *r* = 0.647, *p* = 0.001; Ki67+ *r* = 0.778, *p* = 0.000; FoxP3+ *r* = 0.618, *p* = 0.006).

We then analyzed these significant associations across the clinical-topological subgroups, finding: (1) a significant positive correlation between CD3+ TILs at the tumor center and CD3+ TILs at the tumor margin, in both the relapse (*r* = 0.694, *p* = 0.038) and non-relapse (*r* = 0.632, *p* = 0.020) subgroups; (2) a significant positive correlation between Ki67+ TILs at the tumor center and Ki67+ TILs at the tumor margin, in both the relapse (*r* = 0.858, *p* = 0.001) and non-relapse (*r* = 0.724, *p* = 0.002) subgroups; (3) a significant positive correlation between FoxP3+ TILs at the tumor center and FoxP3+ TILs at the tumor margin, in the non-relapse subgroup (*r* = 0.738, *p* = 0.010).

All significant correlations, between and within TIL subsets, are listed in Table 2.

## 3. Discussion

### 3.1. Quantification and Distribution of TIL Subsets

In our cohort of invasive breast cancer patients, TILs represented the 3.78% on average of the tumor mass, FoxP3+ TILs the 0.55%, and occurred with an equal distribution in the tumor center and margin. A plausible reason why we rarely found Tregs in the analyzed tissues might be the early stage of the investigated breast tumors, mainly T1 N0 M0. In fact, FoxP3+ cells are more often detected in advanced tumor stages with lymph node involvement [40,41], and are likely to be located in the surrounding stroma [25], which we did not consider in the present study. We separately evaluated TILs at the invasive edge and TILs at the center of the tumor, to find any clinical implications of tumor heterogeneity. No statistically significant differences of quantity and distribution of both TIL subsets and TIL ratios were found, either when comparing topological and clinical groups and subgroups.

Although the clinical groups were matched for histological subtype, tumor stage and hormone receptor status, the distribution of the hormone receptor status between the two cohorts was not equal. This factor could represent a limitation for a comparative analysis, given breast cancer heterogeneity and the different impact of the immune infiltrate on outcome across breast cancer subtypes. As such, the presence of TILs was shown to be potentially prognostic in triple-negative breast cancer (TNBC) and human epidermal growth factor receptor 2 (HER2)–positive patients [42]. In those tumor subtypes, higher levels of TILs were observed to be associated with better overall survival and fewer recurrences, independently from the therapy [21,34,43] and the immune subpopulations of the infiltrate [13,34,43,44]. Nevertheless, the functionality of various TILs and their composition should also be taken into account for a complete breast cancer assessment and management [42].

### 3.2. Topological and Clinical TIL Inter-/Intra-Subtype Correlations

Despite the lack of prognostic impact of the quantity and the distribution of the single TIL subsets, we found the analysis of correlations between and within TIL subtypes to be more crucial for understanding how the tumor immune microenvironment differs between relapse and non-relapse patients. Inter-subtype lymphocytic correlations (between CD3-CD8, FoxP3-Ki67, C3FR-C8FR) were significant only in the non-relapse group, while intra-subtype lymphocytic correlations (within CD3, Ki67) were found to be significant in both relapse and non-relapse groups. This may suggest the existence of stronger inter-subtype lymphocytic networks in invasive breast cancer without recurrence, which might have a role in preventing relapse.

In the following, we discuss our findings in the context of the current literature, providing a concise background of those TIL subsets showing significant correlations in our patients.

#### 3.2.1. The Effector TILs

There is already evidence of the positive prognostic impact of both CD3 and CD8 markers in large cohorts of estrogen-negative or triple-negative breast cancer (TNBC) [32,33,34]. Furthermore, CD3 was found to be an independent marker of good prognosis in ductal breast cancer [36] and correlated to a better overall survival [35,45]. Similarly, CD8 was observed to be a strong prognostic factor for risk stratification in breast cancer patients [37], leading to better prognosis, recurrence-free, cancer-specific survival [12,18] and clinical outcome [37,38]. In our previous study [31], the expression of CD3+ and CD8+ lymphocytes had no statistically impact on disease-free and overall survival in invasive ductal breast cancer. Likewise, in our current study, although higher CD3 and CD8 expressions were observed in the non-relapse group, no statistically significant difference of quantity and distribution was found by comparing topological as well as clinical groups. There was, however, a significant positive correlation between CD3+ and CD8+ T-cells at the tumor center in the non-relapse group, suggesting a possible role of CD3-CD8 lymphocytic network in preventing recurrence.

#### 3.2.2. The Suppressor TILs

Findings on FoxP3+ TILs are still not consistent: in our previous work on invasive ductal breast cancer [31], FoxP3 was an independent prognostic factor of disease-free and overall survival, while other studies on mammary cancer observed a connection of high FoxP3 expression with reduced progression-free and overall survival [46,47]. Moreover, a high number of FoxP3+ cells was found to be associated with a poor prognosis, increased invasiveness and probability of metastasis occurrence in several solid tumors [40,48,49,50], such as renal cell carcinoma [51] and ovarian cancer [52]. FoxP3 is also more likely to be an indicator of tumor-induced immune evasion [31], since FoxP3+ cells are responsible for inactivation of tumor-specific immune defense [40,53] and autoreactive T lymphocytes, such as CD8+ cells [19,40,54]. In the present study, a similar FoxP3 expression was observed in both relapse and non-relapse patients. However, the non-relapse group showed a positive intra-subtype TIL correlation between FoxP3+ cells at tumor margin and center, as well as a positive inter-subtype TIL correlation between FoxP3+ and Ki67+ cells. Overall, patients without relapse exhibited several significant correlations of both suppressor (CD3+, CD8+) and effector (FoxP3+) TILs, suggesting that strong lymphocytic networks, independently from their suppressor/effector nature, underpin tumor balance, with a less connected and wired tumor immune microenvironment potentially increasing the risk of relapse.

#### 3.2.3. The Effector to Suppressor TIL Ratios

To have a more complete picture of the tumor immune microenvironment, we also evaluated the ratios (CFRs) of effector T-cells (CD3+, CD8+) to suppressor T-cells (FoxP3+), as prognostic variables. The importance of CFRs as prognostic biomarkers has been shown in previous studies on solid tumors, including breast cancer. Low intraepithelial C3FR was found to be correlated with shorter patient survival time in colon cancer [55] and with adverse outcomes in early-stage non-small cell lung cancer [56]. Similarly, the C8FR was an independent prognostic factor in colorectal tumor and a predictive marker for both disease-free and overall survival times [50]. A lower C8FR was associated with adverse outcome in patients with ovarian cancer [57] and hepatocellular carcinoma [58]; while a higher C8FR had a positive prognostic impact on serous ovarian cancer [59]. Importantly, the C8FR was recently shown to be a valid biomarker also in breast cancer [12,18,19]. It was a useful predictor of treatment response to neoadjuvant therapy in aggressive breast cancer subtypes [19], of relapse of ductal carcinoma in situ (DCIS) [12], and of prognosis in TNBC patients [18]. A lower C8FR was associated with the probability of relapse [12], while a higher C8FR predicted favorable prognosis [18,19]. In the present study, although higher CFRs were observed in the non-relapse group, no statistically significant difference of quantity and distribution was found by comparing topological as well as clinical groups. This may mean that the presence of both effector and suppressor TILs was similar in tumor margin and center, in relapse and non-relapse conditions. Nevertheless, a strong tumor heterogeneity was observed in the significant TIL correlations exhibited by the non-relapse group: CD3+ and CD8+ lymphocytic infiltrations were found to be positively inter-correlated at the tumor center, while C3FR and C8FR showed a positive correlation at the invasive edge of the tumor. These findings might be indicative of the presence of two different protective networks against relapse: a protective “effector” TIL network (CD3–CD8) at the tumor center, as well as a protective “effector/suppressor” TIL balance (C3FR–C8FR) at the tumor margin, possibly relevant to keep relapse-initiating CSCs and EMT dormant.

## 4. Materials and Methods

### 4.1. Cohort and Sample Selection

In this retrospective study, patients were selected among those treated for invasive breast cancer, between July 2008 and September 2009, at the Breast Cancer Center of the Department of Gynecology and Obstetrics, University Hospital Schleswig-Holstein, Campus Kiel, Germany. Written informed consent was available for all patients and approved by the ethics committee.

Carcinomas were classified according to the criteria of the World Health Organization. Staging at the time of diagnosis was based on the TNM (tumor, node and metastasis) system [60]. The selection criteria were: (a) tumor size and (b) availability of high-quality formalin-fixed paraffin-embedded tissue (FFPE). Tumors less than 2 cm in diameter were included in the study, to reliably distinguish between tumor center and tumor margin/invasion front on one full slide of the tumor. Thirteen patients out of those registered at the Breast Cancer Database fulfilled the selection criteria and were included in the relapse group, suffering from a local or distant recurrence of the tumor.

Eighteen patients were selected for the non-relapse group, suffering from an invasive breast cancer without showing local or distant recurrence of the tumor during a median follow-up of 54 months (range 36–132 months). They matched the relapse group by histological subtype, tumor stage and receptor expression (estrogen and progesterone receptors’ expression ≥ score 3), as shown in Table 3.

None of the patients in the relapse or non-relapse groups underwent preoperative radiation or chemotherapy. All patients received appropriate postoperative treatment depending on the stage of the disease, including chemotherapy, radiation and medical anti-estrogen therapy, when indicated.

The clinical parameters and prognostic factors (tumor staging; histological type; tumor grading; hormone receptor status: estrogen receptor, progesterone receptor, Her2neu status) of the relapse and non-relapse groups are outlined in Table 3, where absolute as well as relative frequencies are provided.

DCIS, normal breast tissue adjacent to the tumor as well as tissue from breast reduction were analyzed as further controls.

### 4.2. Tissue Micro Arrays (TMA)

FFPE specimens were retrieved from the archives of the Department of Pathology. Histological examination was performed with hematoxylin and eosin staining (H&E) and representative areas were selected and assembled in a tissue microarray (TMA), using cores of 1.0 mm diameter and a TMA1 Tissue Arrayer (Beecher Instruments, Sun Prairie, WI, USA). Areas in the tumor center and the invasion front were selected and punched independently, with the distance between both areas being >2 mm.

### 4.3. Immunohistochemistry

Three µm sections of the TMA were used for immunohistochemistry. Antigen retrieval was performed for the FoxP3 antibody manually, with an EDTA buffer pH8 for 3 min, by boiling in a pressure cooker. The primary antibody was applied for one hour at room temperature (mouse, monoclonal FoxP3 antibody, 1:250, pH8, Abcam, Cambridge, UK). The secondary antibody (Histofine: Simple MAX PO (Multi) Universal Immuno-peroxidase Polymer produced by Medac, (Chicago, IL, USA) was applied for 30min at room temperature. The detection was performed using 100 µL/slide Dako DAB (Agilent, Santa Clara, CA, USA).

The antibodies for CD3 (rabbit, monoclonal CD3 antibody, 1:100, pH6, NeoMarkers, (Portsmouth, NH, USA), CD8 (mouse, monoclonal CD8 antibody, 1:100, pH6, Dako), CD20 (mouse, monoclonal CD20 antibody, 1:5, pH6, own production), Ki67 (mouse, monoclonal Ki67 antibody, 1:5, pH6, own production) and pan-keratin (mouse, monoclonal pan-keratin antibody, 1:200, pH8, NeoMarkers) were applied by the Bond MAX system of Leica and the detection system Bond Polymer Refine Detection (Leica Biosystems Newcastle, United Kingdom, catalog No: DS9800). Firstly, the tissues were incubated in hydrogen peroxide to quench endogenous peroxidase activity; then, the antigen retrieval was either done with a citrate buffer pH6 or with pH8 EDTA buffer. The incubation of the primary antibody with the Bond MAX system takes 15 min. A post primary IgG linker is used to detect the primary antibody. Subsequently a Poly-HRP IgG reagent localizes the antibody complex for heightening the staining intensity. Furthermore, in the automatic staining 3,3′-diaminobenzidine tetrahydrochloride (DAB) and hematoxylin counterstaining were used for the visualization. For negative controls, the primary antibodies were omitted. The tissue was analyzed by light microscopy (Zeiss Axiophot, Zeiss GmbH, Jena, Germany) and reviewed by ProCapture software (Mawson Lakes, South Australia). Only positive stained cells in a cluster of cancer cells were assessed and counted manually, to determine the percentage of lymphatic cells in the tumor. 100 cells of a tumor cluster in every TMA Core were counted and the number of containing lymphatic cells was determined. Data on hormone receptor status were scored following immunohistochemistry staining guidelines of the American Society of Clinical Oncology and College of American Pathologists [61,62].

### 4.4. Ethics Statement

This study was approved by the Ethical Committee of the Christian-Albrechts-University Kiel, Kiel, Germany (D 426/10; 31 August 2010). The board chairman is Professor H.M. Mehdorn and the managing director is C. Glienicke. All the living patients signed an informed consent to allow the use of their tumor specimen and clinical data.

### 4.5. Statistical Analysis

All statistical analyses were carried out in SPSS^®^ version 23 statistical software (IBM, Armonk, NY, USA). Group comparisons were done by performing independent-samples *t*-tests. Correlations between and within variables were revealed by running bivariate Pearson Correlation. Cases with missing values were excluded on an analysis-by-analysis basis (pairwise deletion).

## 5. Conclusions

In evaluating TILs in invasive breast cancer, quantity and distribution of single TIL subsets did not show any prognostic impact on our patients. In contrast, we found statistically significant correlations between and within TIL subtypes to be indicative of the extent to which the tumor immune microenvironment differed between relapse and non-relapse conditions.

(1) Patients without relapse exhibited several significant correlations of both suppressor (CD3, CD8) and effector (FoxP3) TILs, suggesting that the presence of strong lymphocytic networks might have a role in maintaining the tumor lymphocytic balance, meaning that a less wired and connected tumor immune microenvironment might be more prone to relapse.
(2) Inter-subtype lymphocytic correlations (between CD3–CD8, FoxP3–Ki67, C3FR–C8FR) were significant only in the non-relapse group, while intra-subtype lymphocytic correlations (within CD3, Ki67) were found to be significant in both clinical groups. This may suggest that in particular the presence of strong inter-subtype lymphocytic networks might play a role in preventing breast cancer recurrence.
(3) Moreover, the non-relapse group exhibited tumor heterogeneity in terms of distribution of lymphocytic networks. In fact, a significant positive correlation was found between CD3+ and CD8+ T-cells at the tumor center, whereas C3FR and C8FR were found to be positively correlated at the invasive edge of the tumor. This may suggest the presence of two different protective networks: a protective “effector” TIL network (CD3–CD8) at the tumor center, as well as a protective “effector/suppressor” TIL balance (C3FR–C8FR) at the tumor margin, possibly to control relapse-initiating CSCs and EMT.

Further evaluations of clinical and topological correlations between and within TIL subsets are needed, in addition to TIL quantification and distribution, to further investigate whether morphological evaluation of TILs might reveal the underlying tumor lymphocytic connectivity. A deeper understanding of breast tumor immunogenicity and lymphocytic networks can shed new light on tumor progression mechanisms, further the development of more effective prognosis techniques and improve treatment responses.

## Figures and Tables

**Table 1:** Arithmetic average in percent (%) of TIL subpopulations (CD3+, CD8+, CD20+, FoxP3+, Ki67+) and ratios (CD3/FoxP3, CD8/FoxP3) in invasive breast cancer samples. All the compared groups and subgroups are listed with the respective *p*-value underneath.

Invasive Breast Cancer Samples	CD3	CD8	CD20	FoxP3	Ki67	CD3/FoxP3	CD8/FoxP3
All samples (*n* = 62)	3.8	1.58	0.15	0.54	11.77	3.73	1.78
Clinical groups							
R (*n* = 26)	3.67	1.06	0.1	0.58	12.46	3.6	0.82
N (*n* = 36)	3.88	1.98	0.18	0.52	11.31	3.82	2.74
	*p* 0.825	*p* 0.137	*p* 0.447	*p* 0.801	*p* 0.753	*p* 0.886	*p* 0.334
Topological groups							
M (*n* = 31)	4.27	1.82	0.07	0.69	10.84	3.14	2.31
C (*n* = 31)	3.26	1.27	0.23	0.38	12.81	4.74	1.02
	*p* 0.263	*p* 0.409	*p* 0.127	*p* 0.232	*p* 0.580	*p* 0.298	*p* 0.524
Subgroups							
RM (*n* = 13)	4.02	1.14	0	0.84	11.42	2.7	0.76
NM (*n* = 18)	4.45	2.32	0.12	0.6	10.46	3.46	3.63
	*p* 0.882	*p* 0.245	*p* 0.163	*p* 0.560	*p* 0.844	*p* 0.620	*p* 0.401
RC (*n* = 13)	3.3	0.98	0.2	0.33	13.59	5.11	0.9
NC (*n* = 18)	3.23	1.5	0.25	0.42	12.28	4.46	1.29
	*p* 0.944	*p* 0.521	*p* 0.779	*p* 0.806	*p* 0.816	*p* 0.855	*p* 0.634

R = relapse group; N = non-relapse group; M = tumor margin; C = tumor center; RM = relapse tumor margin; NM = non-relapse tumor margin; RC = relapse tumor center; NC = non-relapse tumor center; *n* = number. The *p*-value is significant when <0.05 (no significant *p*-values are shown in Table 1).

**Table 2:** Significant correlations between different TIL subsets (inter-subtype correlations) and within same TIL subsets (intra-subtype correlations) in invasive breast cancer, with their respective *r* coefficient, *p*-value and sample size.

Significant Correlations	Pearson Correlation (*r* Coefficient)	Significance (2-Tailed) Value	Correlation’s Sample Size
Inter-subtype correlations			
CD3–CD8	0.392	0.009 **	43
CD3c–CD8c	0.496	0.031 *	19
CD3n–CD3n	0.469	0.016 *	26
FoxP3–Ki67	0.337	0.024 *	45
FoxP3c–Ki67c	0.803	0.000 **	21
FoxP3c–Ki67m	0.457	0.043 *	20
FoxP3n–Ki67n	0.55	0.003 **	27
FoxP3nc–Ki67nc	0.887	0.000 **	12
FoxP3nm–Ki67nc	0.582	0.037 *	13
C3FR–C8FR	0.56	0.013 *	19
C3FRm–C8FRm	0.884	0.000 **	12
C3FRnm–C8FRnm	0.911	0.004 **	7
Intra-subtype correlations			
CD3c–CD3m	0.647	0.001 **	22
CD3rc–CD3rm	0.694	0.038 *	9
CD3nc–CD3nm	0.632	0.020 *	13
Ki67c–Ki67m	0.778	0.000 **	26
Ki67rc–Ki67rm	0.858	0.001 **	10
Ki67nc–Ki67nm	0.724	0.002 **	16
FoxP3c–FoxP3m	0.618	0.006 **	18
FoxP3nc–FoxP3nm	0.738	0.010 **	11

C3FR = CD3/FoxP3; C8FR = CD8/FoxP3; marks specify various clinical and/or topological groups/subgroups: r = relapse; n = non-relapse; m = tumor margin; c = tumor center; rm = relapse tumor margin; nm = non-relapse tumor margin; rc = relapse tumor center; nc = non-relapse tumor center; when no mark is specified, we refer to the whole sample. * Correlation is significant at the 0.05 level (2-tailed). ** Correlation is significant at the 0.01 level (2-tailed).

**Table 3:** Clinicopathological parameters and of the relapse (R) and non-relapse (NR) group.

Parameters	R Group’s Absolute Frequency (*n* = 13)	R Group’s Relative Frequency %	NR Group’s Absolute Frequency (*n* = 18)	NR Group’s Relative Frequency %
TNM classification				
T1	13	100	18	100
N0	10	76.9	18	100
M0	11	84.6	16	88.9
Histological type				
Ductal	8	61.5	14	77.8
Lobular	2	15.4	3	16.7
Other	3	23.1	1	5.6
Tumor grade				
≤G2	8	61.5	12	66.7
Receptor expression				
ER+ ≥ 3	7	53.8	15	83.3
PR+ ≥ 3	6	46.1	12	66.7
Her2neu ≥ 2	2	15.4	4	22.2
Patients’ age				
Mean	51		55	
Max	68		72	
Min	36		36	
Time of follow-up *	99		54	

R = relapse; NR = non-relapse; T1 = T1 stage (tumor size ≤ 2 cm across); N0 = N0 stage (no cancer cells in any nearby nodes); M0 = M0 stage (no distant metastasis); ≤G2 = grade 2 or 1 (well/moderately differentiated cancer cells); ER+ ≥ 3 = estrogen receptor expression ≥ score 3; PR+ ≥ 3 = progesterone receptor expression ≥ score 3; *n* = total number; * median of follow-up duration in months.
